# Characterization of a novel mCH3 conjugated anti-PcrV scFv molecule

**DOI:** 10.1038/s41598-021-86491-w

**Published:** 2021-03-30

**Authors:** Samira Komijani, Elham Bayat, Elham Rismani, Soma Hosseini, Reza Moazzami, Leila Nematollahi, Soroush Sardari, Yeganeh Talebkhan, Fatemeh Davami, Farzaneh Barkhordari, Fakhrisadat Hosseini, Hoda Jahandar

**Affiliations:** 1 Department of Biotechnology School of Biology, Alzahra University, Tehran, Iran; 2 Department of Molecular and Cellular Sciences, Faculty of Advanced Sciences & Technology, Tehran Medical Branch, Islamic Azad University, Tehran, Iran; 3 Molecular Medicine Department, Biotechnology Research Center, Pasteur Institute of Iran, Tehran, Iran; 4 Pharmaceutical Sciences Research Center, Tehran Medical Sciences, Islamic Azad University, Tehran, Iran; 5 Department of Basic Sciences, Faculty of Pharmacy and Pharmaceutical Sciences, Tehran Medical Sciences, Islamic Azad University, Tehran, Iran; 6 Present Address: Biotechnology Research Center, Pasteur Institute of Iran, No.358, 12 Farvardin St., 1316943551 Tehran, Iran

**Keywords:** Biological techniques, Biotechnology

## Abstract

*Pseudomonas aeruginosa* (PA) is a leading cause of nosocomial infections and death in cystic fibrosis patients. The study was conducted to evaluate the physicochemical structure, biological activity and serum stability of a recombinant anti-PcrV single chain variable antibody fragment genetically attached to the mCH3cc domain. The stereochemical properties of scFv-mCH3 (YFL001) and scFv (YFL002) proteins as well as molecular interactions towards *Pseudomonas aeruginosa* PcrV were evaluated computationally. The subcloned fragments encoding YFL001 and YFL002 in pET28a were expressed within the *E. coli* BL21-DE3 strain. After Ni–NTA affinity chromatography, the biological activity of the proteins in inhibition of PA induced hemolysis as well as cellular cytotoxicity was assessed. In silico analysis revealed the satisfactory stereochemical quality of the models as well as common residues in their interface with PcrV. The structural differences of proteins through circular dichroism spectroscopy were confirmed by NMR analysis. Both proteins indicated inhibition of ExoU positive PA strains in hemolysis of red blood cells compared to ExoU negative strains as well as cytotoxicity effect on lung epithelial cells. The ELISA test showed the longer serum stability of the YFL001 molecule than YFL002. The results were encouraging to further evaluation of these two scFv molecules in animal models.

## Introduction

*Pseudomonas* *aeruginosa* (PA) is an opportunistic pathogenic bacterium which can cause acute and chronic infections in immunocompromised individuals where the physical barriers of the body have been damaged^1,2^. Several studies have shown PA resistance towards a wide range of antibiotics such as cephalosporins, aminoglycosides, and quinolones^3^. The Type III secretion system (T3SS) is one of the virulence factors involved in transportation of PA effector molecules and toxins (ExoY, ExoT, ExoS, and ExoU) into the cytoplasm of the host cells^4,5^. PcrV, a hydrophilic protein (~ 32 kDa), forms the Type III secretion system needle tip complex which is required for proper accumulation of PopD and PopB proteins^6^.

Due to the high frequency of multidrug-resistant *P. aeruginosa* strains, development of antibody-based treatment approaches has attracted much attention during the last decades^7,8^. In 2002, the first mouse IgG2b monoclonal antibody (Mab166) was developed against the PcrV molecule representing neutralization of the PA infection in a mouse model^1,9^. Although highly specific monoclonal antibodies have been favored for research and clinical applications in recent years, using traditional hybridoma approaches in the generation of human monoclonal antibodies are still tedious and expensive processes for therapeutic purposes^9,10^. In 2009, Baer et al. engineered a human Fab antibody fragment against PcrV protein which competed with MAb166 in binding to the same epitopes^11^. This Fab molecule showed significant potency in in vitro and in vivo TTSS-neutralizing activity very similar to that of the Mab166 against lethal doses of PA.

KB001 is a PEGylated recombinant human Fab antibody fragment designed against PcrV^12^. This antibody represented potential activity in a model of mouse pulmonary infection in which reduced mortality and effective clearance of bacteria was observed within the infected lungs. In order to identify an anti-PcrV MAb possessing enhanced inhibition of the T3SS, Warrener et al. developed a panel of novel antibodies amongst which 29D2 and V2L2MD provided the strongest protection against *P. aeruginosa* infection in an animal model^13^.

Previous studies have shown that the interaction of the IgG1 Fc constant region with the neonatal Fc receptor (FcRn) presented on dendritic cells, neutrophils and epithelium of the kidney plays a critical role in maintaining the long half-life of antibody molecules^14,15^. The importance of CH3 domain in this process has been well documented^16,17^. The successful production of a soluble, monomeric CH3 domain (mCH3) was reported in 2013 in which pH-dependent binding to FcRn was similar to that of Fc moiety^18^. Single-chain variable fragment antibodies (scFv) have been composed of variable regions of the heavy (VH) and light (VL) chains of antibodies linked together by a short peptide linker^19^. The aim of the present study was to evaluate the physicochemical and biological properties of the recombinantly mCH3 conjugated anti-PcrV scFv molecule in in vitro conditions.

## Results

### Design of the constructs

**Figure 1:**
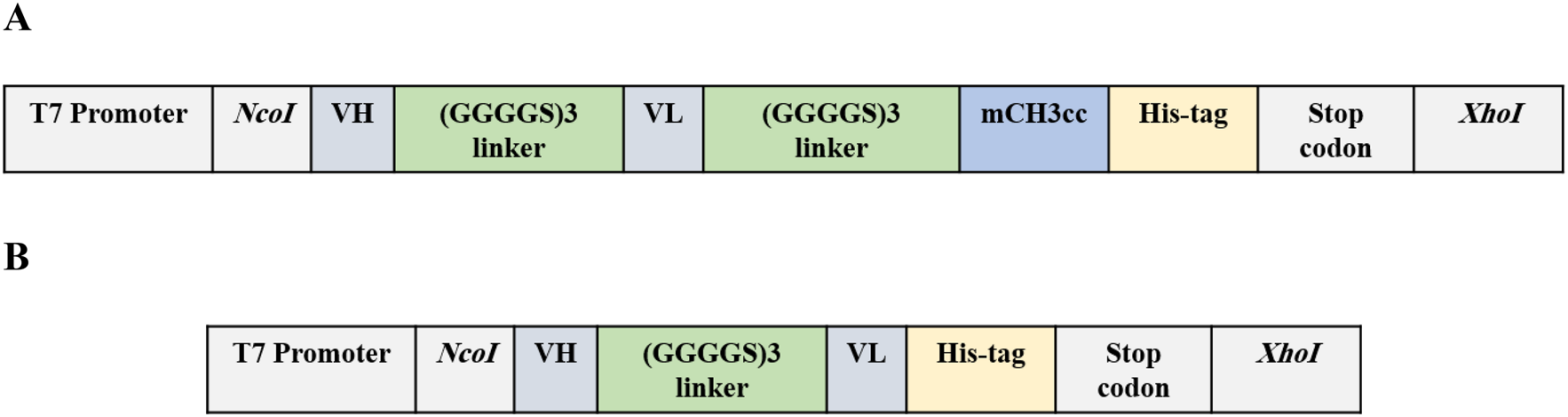
Schematic view of the expression cassettes: (**A**) YFL001; (**B**) YFL002.

YFL002 encoding gene fragment was cloned in *NcoI* and *XhoI* sites of the pET28a expression vector. In another experiment, a mCH3 encoding gene fragment was inserted at 5′ end of anti-PcrV scFv and the YFL001 expression cassette was designed (Fig. 1A,B). The identity of the expression vectors was confirmed through restriction digestion and sequencing analysis (data not shown).

### Validation of 3D YFL001 and YFL002 structural models

**Figure 2:**
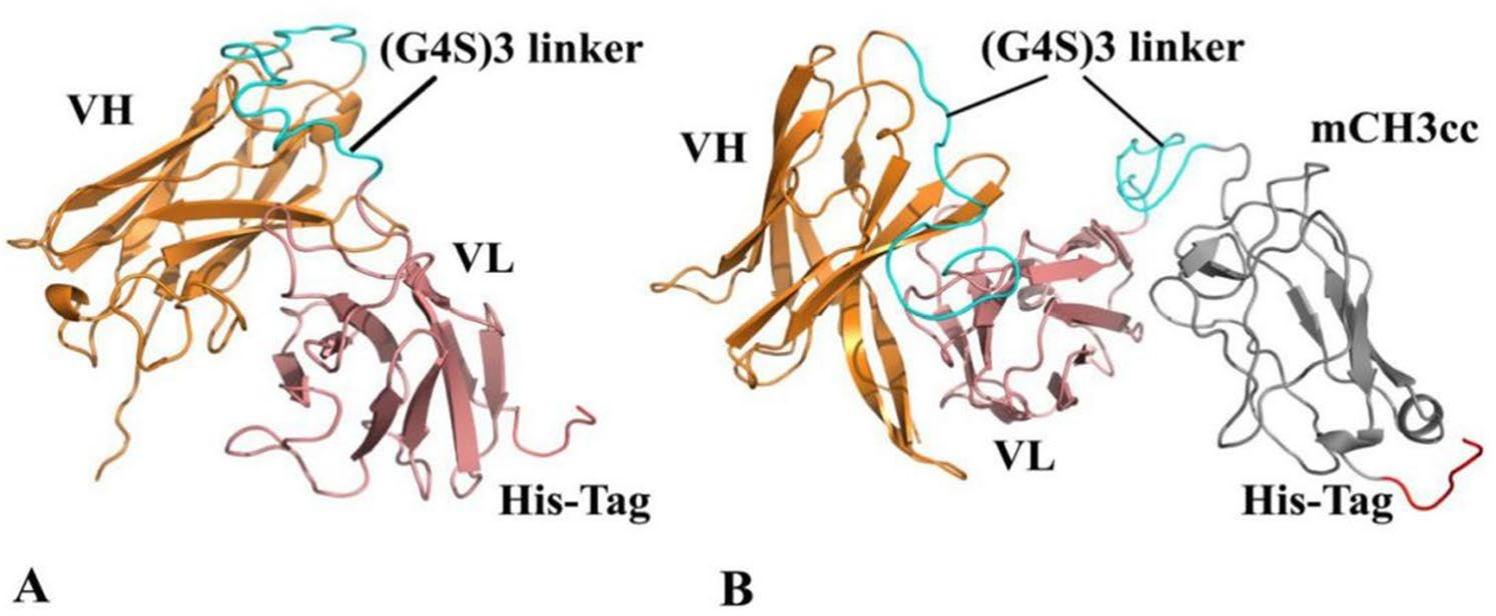
Cartoon representation of predicted 3D structures: **(A)** YFL002; (**B)** YFL001. VH, VL, linker, mCH3cc and His tag are shown in orange, pink, cyan, gray and red, respectively.

MODELLER predicted the 3D structures of YFL001 and YFL002 with knowledge-based constraints. The cartoon representation of the top protein models was depicted in Fig. 2. The stereochemical quality of the top predicted structures was evaluated using several methods. Concerning the topological similarity of the two proteins, TM-score is a metric of the global fold similarity. It is measured as a value in the range of (0,1], where one denotes a perfect match between two structures. The pairwise comparison of the structural models towards the template 3D structures revealed the folding similarities with the score of 0.97 and 0.81 for VH-VL of YFL001 and YFL002, respectively. A score higher than 0.5 is generally assumed for the same two structural folds (Table 1).

**Table 1:** Structural validation of predicted models.

Proteins	TM-Score	ProSA^1^ Z-score	Ramachandran plot quality (%)				Verify 3D (%)^2^	ERRAT (%)^2^
			Most favored	Additionally allowed	Generously allowed	Disallowed		
YFL001	0.97/0.81*	− 6.4	87	9.7	1.3	1.9	84.54	75.62
YFL002	0.97	− 6.4	92.1	5.4	2	0.05	91.76	72.32

^1^ProSA Z-score determines the overall model quality.

^2^100 is the best and 0 is the worst.

*TM-scores of the pairwise structural comparison of YFL001 fragment towards the templates (VH-VL compared to 6CYF.LM and mCH3cc compared to 5HSF.A, respectively).

The overall quality of the models, computed by ProSA Z-score, indicated the value within the range experimentally calculated for protein structures by X-ray and NMR (Table 1). PROCHECK Ramachandran plots showed the values of the most favored region of 87% and 92.1% for YFL001 and YFL002, respectively. It revealed that stereochemical quality of the models was satisfactory and an acceptable percentage of amino acid residues were located in the most favored region.

The compatibility of the 3D models was analyzed by Verify3D based on the amino acid sequence. A well compatible 3D structure is defined when more than 80% of the amino acids are scored at more than 0.2. The Verify3D score of YFL001 and YFL002 has passed the acceptable range with the values of 84.54% and 91.76%, respectively. Finally, the non-bonded atomic interactions of the models were assessed based on the ERRAT value as an overall quality factor in which a score above 50% represents the reliability of the model. The values of 75.62% and 72.32% indicated the reliable predicted structures of YFL001 and YFL002, respectively (Table 1).

### Physicochemical properties of the two recombinant proteins

The calculated isoelectric point (pI) of YFL001 and YFL002 proteins (6.69 and 7.08, respectively) revealed their neutral behavior where the overall charge of the proteins was zero. The lower GRAVY values reflect the characteristics of a soluble protein whereas the more positive values are indicative of the hydrophobic proteins. The estimated half-life of YFL001 and YFL002 proteins was also calculated by ProtParam (Table 2).

**Table 2:** Physicochemical properties of the proteins by ProtParam.

Proteins	Length (a.a)	pI	GRAVY^1^	Estimated half-life (hours)		
				Mammalian reticulocytes (in vitro)	Yeasts (in vivo)	*E. coli* (in vivo)
YFL001	388	6.69	− 0.476	30	> 20	> 10
YFL002	261	7.08	− 0.263	30	> 20	> 10

^1^Grand average of hydropathicity.

### Interaction analysis of YFL001 and YFL002 antibodies towards PcrV antigen

**Figure 3:**
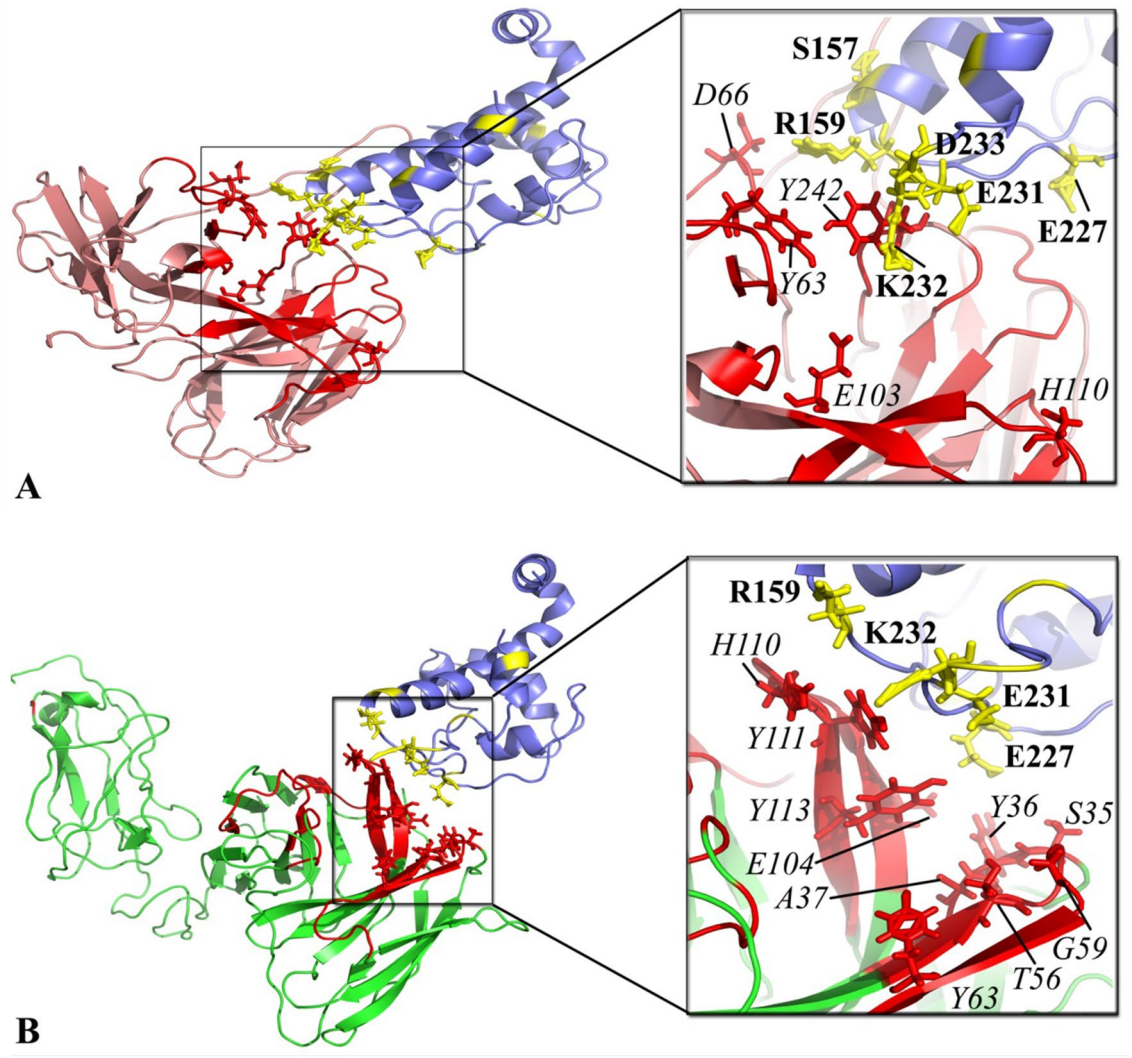
Molecular docking of scFv proteins towards PcrV: **(A)** YFL002/PcrV complex, **(B)** YFL001/PcrV complex. PcrV, YFL001, and YFL002 are shown in blue, green, and pink colors, respectively. CDRs of anti-PcrV molecules and binding residues of PcrV are in red and yellow, respectively. The interacting residues of YFL001, YFL002 and PcrV are in sticky format where YFL001 and YFL002 residues are italic and PcrV residues are bolded.

To determine the interaction mode of YFL001 and YFL002 towards PA PcrV antigen, molecular docking was performed by defining the interface amino acid residues located within the scFv CDRs and PcrV antigen. Binding pattern analysis of the complexes revealed that relatively similar residues were involved in the interaction of these two scFv molecules towards PcrV (Fig. 3). However, higher number of active residues was detected within the complex of YFL001/PcrV.

### Molecular dynamic interactions of scFv/PcrV

**Figure 4:**
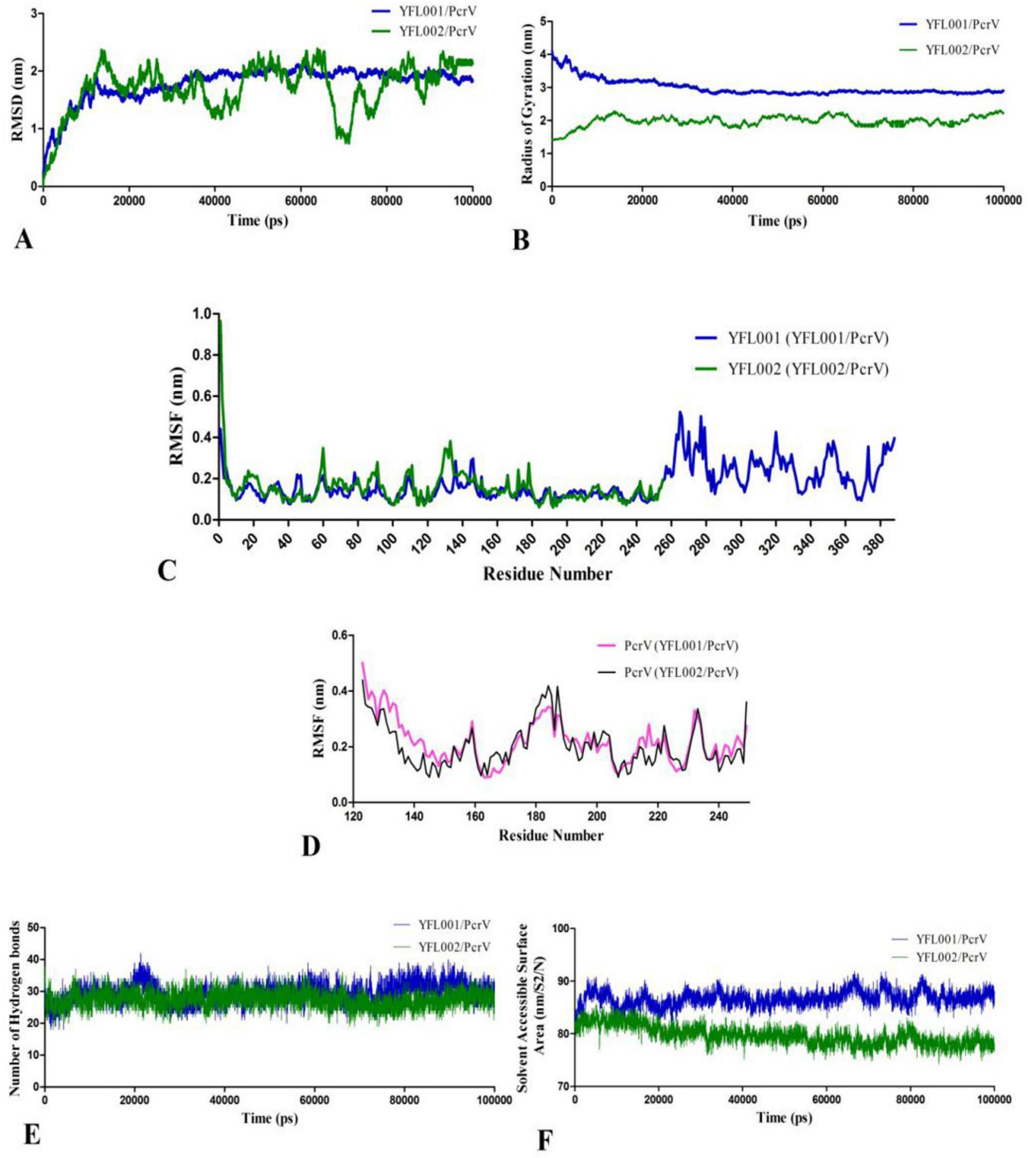
MD simulations of YFL001/PcrV and YFL002/PcrV complexes: **(A)** RMSD; (**B**) RoG; (**C**) RMSF values of YFL001 and YFL002 molecules; (**D**) RMSF values of PcrV antigen; (**E**) Number of H-bonds; (**F**) SASA plots. Plots of YFL001 and YFL002 in their complexes with PcrV are shown in blue and green, respectively. Plots of PcrV within the complexes are in pink and black, respectively.

The docking complexes of YFL001/PcrV and YFL002/PcrV were subjected to 100 ns MD simulations using GROMACS package. The MD output trajectories were analyzed in terms of root mean square deviation (RMSD) of the structures during simulations. The RMSD value against the simulation period (100 ns) is depicted in Fig. 4A. The plot showed higher fluctuations in YFL002/PcrV complex compared to the YFL001/PcrV. Although both complexes have converged towards an equilibrium state at a final 10 ns of simulations, YFL001/PcrV complex displayed the minimum deviation during simulations. It can be interpreted as higher conformational stability of YFL001/PcrV complex compared to YFL002/PcrV. Fewer changes have also been shown in the radius of the gyration plot of YFL001/PcrV indicating a higher compactness and folded state of this complex in comparison to the YFL002/PcrV (Fig. 4B).

The root mean square fluctuations (RMSF) of the alpha carbons were plotted as the average fluctuation of each residue during simulations. The results were displayed for YFL001, YFL002 and PcrV to depict residual fluctuations of proteins in each complex separately. The RMSF values indicate the flexibility of each residue which is comparable to X-ray B-factors^20^.

Amino acid residues of 1 to 255 in YFL001 and YFL002 (encoding the scFv molecule) showed an almost identical fluctuation pattern while residues encoding the mCH3cc fusion fragment illustrated higher fluctuations in YFL001 molecule (Fig. 4C) which was limited in the range of 0.1–0.6 nm. Interestingly, the RMSF plot of PcrV amino acid residues was significantly similar in both complexes (Fig. 4D). Furthermore, formation of a remarkably similar number of H-bonds (in the range of 30 H-bonds) was evaluated between scFv fragment and PcrV molecule during MD simulations within the two complexes (Fig. 4E).

The solvent accessible surface area (SASA) of a biomolecule is the surface area that is attainable to a solvent. High score is considered when more molecules protrude from the solvent while lower scores mean that more molecules are buried within the complex. SASA analysis of the complexes demonstrated that YFL001 had higher accessible surface area. The average SASA values for YFL001/PcrV and YFL002/PcrV remained around 88 and 78 (nm/S2/N), respectively, whereas the SASA values for the interface of YFL001/PcrV and YFL002/PcrV were almost similar (Fig. 4F). Binding energy (ΔG) and dissociation constant (K_d_) of the average stable complexes were extracted from MD trajectories using the PRODIGY server. The results revealed that YFL001 and YFL002 interacted with PcrV with similar free energy of binding (-10 and -11.1 kcal mol^−1^, respectively) and binding affinity (8.60E-08 and 1.40E-08, respectively).

### Protein expression, identification and purification

**Figure 5:**
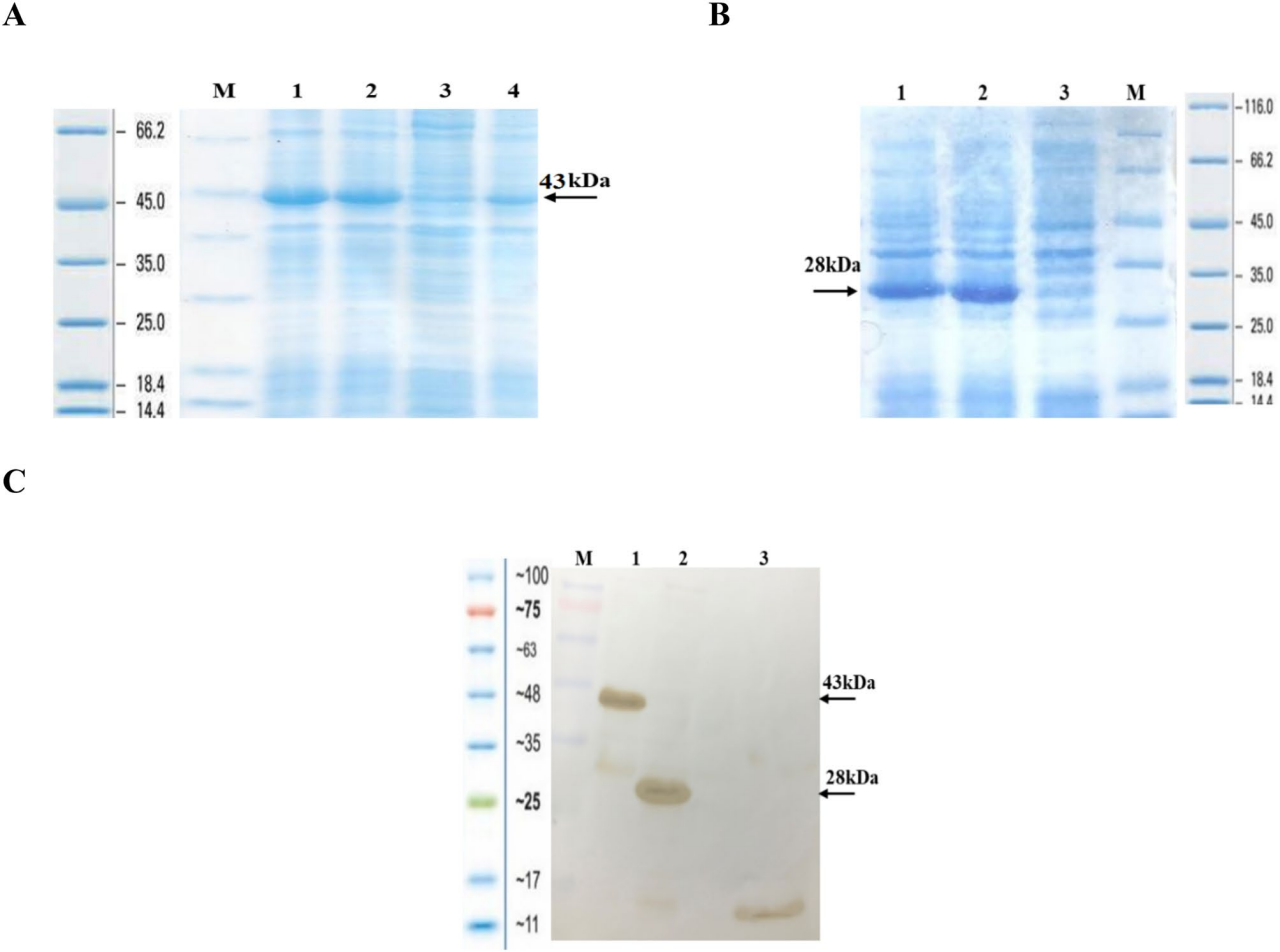
Protein expression analysis: **(A)** YFL001: M: Protein Mw marker (Fermentas); #1, 2, 4: Recombinant *E. coli* lysate after induction (AI); #3: Recombinant *E. coli* lysate before induction (BI); (**B**) YFL002: #1, 2: Recombinant *E. coli* lysate (AI); 3: Recombinant *E. coli* lysate (BI); M: Protein Mw marker (Fermentas); **(C)** Non-reduced Western blotting analysis: M: Protein Mw marker (Thermo Fisher Scientific); #1, 2: YFL001 and YFL002 expressing recombinant *E. coli* lysates (AI); #3: His-tagged 12 kDa recombinant protein (positive control). The full size original gels and the blot are presented in Supplementary Figs. S1, S2 and S3, respectively.

SDS-PAGE analysis represented two recombinant proteins in the range of 43 and 28 kDa (YFL001 and YFL002, respectively) (Fig. 5A,B). Identity of the recombinant proteins was confirmed by Western blotting (Fig. 5C) in which expected protein bands represented monomeric forms of the molecules in non-reduced conditions.

**Figure 6:**
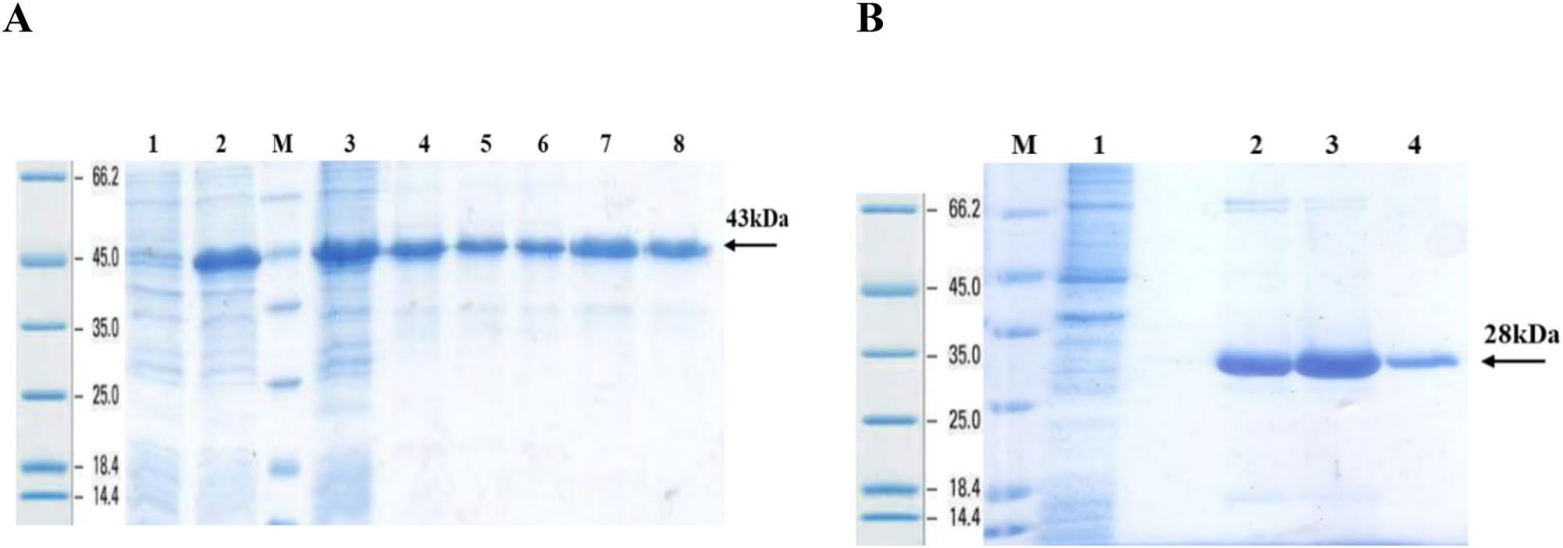
Protein purification: (**A)** YFL001: #1, 2: Recombinant *E. coli* lysate (BI and AI); M: Protein Mw marker (Fermentas); #3: Initial sample; #4–8: Eluted protein samples; (**B)** YFL002: M: Protein Mw marker (Fermentas); #1: Flow through sample; 2–4: Eluted protein samples. The full size original gels and the blot are presented in Supplementary Figs. S4 and S5, respectively.

Initial experiments represented that the r-proteins have been expressed as cytoplasmic inclusion bodies (IBs) (data not shown). Large scale protein expression experiments were performed in 250 ml culture medium and the proteins were purified by the denaturing pH dependent Ni–NTA affinity chromatography approach (Fig. 6A,B).

The polydispersity index (PDI) and hydrodynamic radius measurements of refolded proteins by Dynamic Light Scattering analysis (DLS) revealed that no protein aggregation was formed during refolding procedure (YFL001: 0.39, 123.4 nm; YFL002: 0.4, 115.4 nm, respectively) where the PDI values lower than 0.5 indicate the uniformity of the sample particles^21^.

### Protein structural analysis

**Figure 7:**
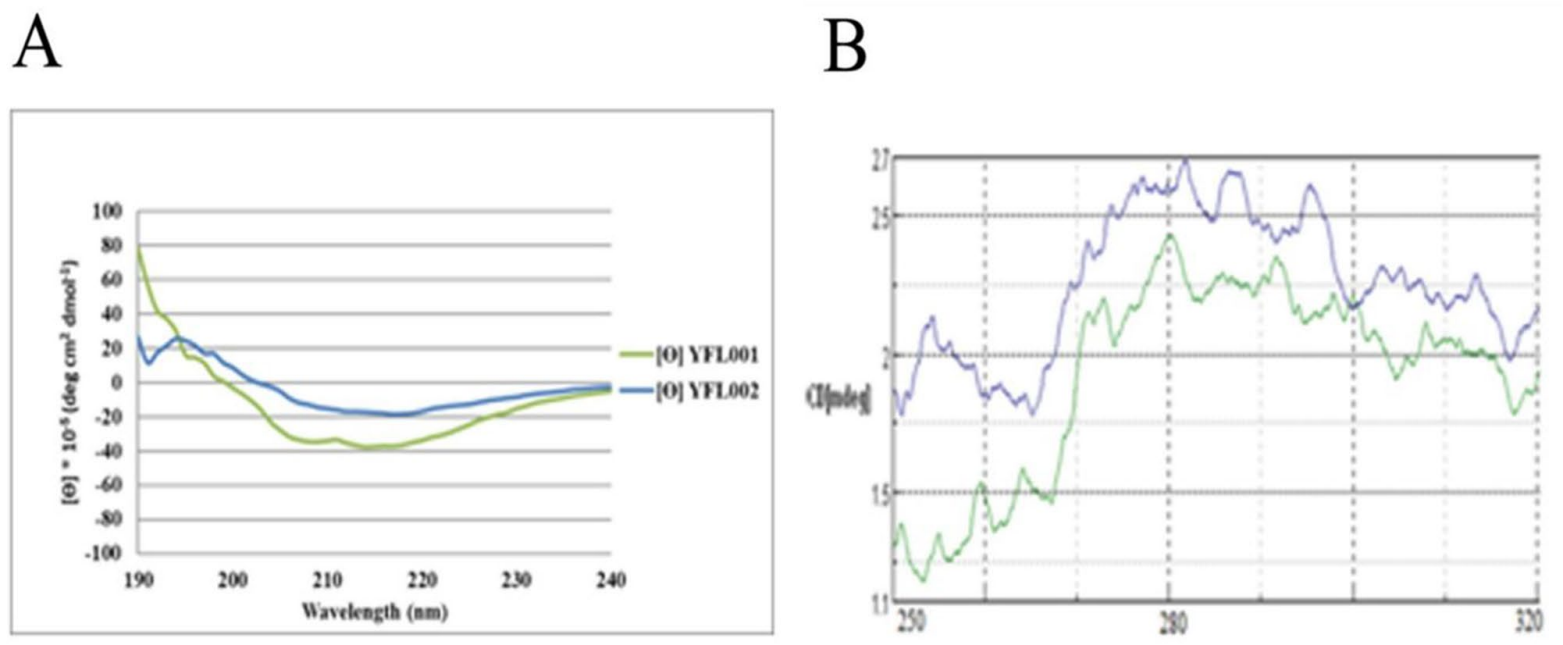
CD structural analysis of the recombinant proteins: **(A)** Far-UV spectra; (**B)** Near-UV spectra (YFL001 in green and YFL002 in blue, respectively).

The results of far and near UV CD spectra to predict and analyze the secondary and tertiary structures of the two purified proteins have been shown in Fig. 7A,B and Table 3.

**Table 3:** Prediction CD spectroscopy of recombinant proteins.

Proteins	Predicted secondary structures	%
YFL001	Alpha helix (H)	25.2
	Extended strand (E)	31.6
	Beta-turn (T)	3.9
	Random coil (C)	39.3
YFL002	Alpha helix (H)	33.9
	Extended strand (E)	30.7
	Beta-turn (T)	13.7
	Random coil (C)	21.6

**Figure 8:**
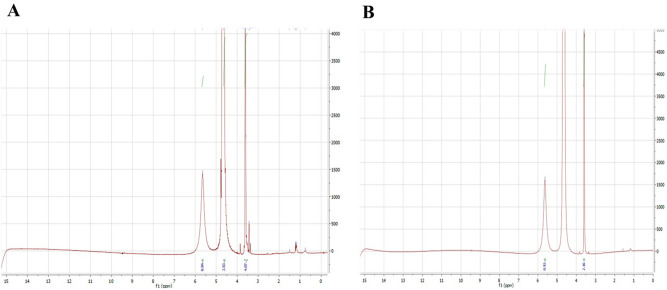
NMR analysis of recombinant proteins: **(A)** YFL001; (**B)** YFL002. Spectral data was analyzed using MestreNova software.

Analysis of the H-NMR spectra using MestreNova software revealed that the overall pattern obtained integration (the total area of peaks corresponding to hydrogens in the protein) from YFL001 and YFL002 was 86.99 and 66.99, respectively indicating the larger size of the YFL001 molecule (Fig. 8). The chemical shifts were extracted from the protein NMR table (available on www.triton.iqfr.es) and compared with H-NMR peaks obtained in this study. A slight shift of some particular amino acids such as arginine, methionine, lysine and aspartic acid was observed in YFL001 which may be due to the differences in protein folding patterns because of the mCH3 incorporation into this molecule.

Proton chemical shifts of the amino acids obtained from the NMR peaks were comparable with the protein NMR table (available on www.triton.iqfr.es). The slight shift appeared in the NMR peaks may be due to the different folding characteristics of the two recombinant molecules (Table 4).

**Table 4:** Proton amino acid shifts by NMR.

Proteins	Val	Ser	Pro	Glu	Ile
YFL001	4.19	3.83	3.66	2.05	1.16
YFL002	4.15	3.86	3.67	2.08	1.19

### Biological activity of the recombinant proteins

#### Inhibition of PA induced RBC cell lysis

**Figure 9:**
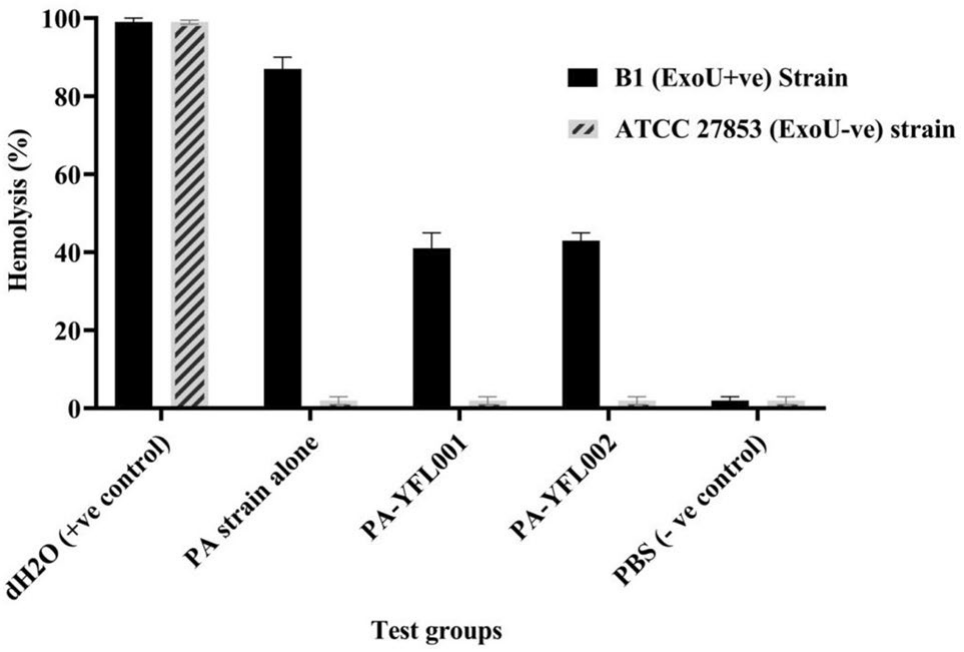
RBC hemolysis. The observed hemolysis activity in the presence and absence of the recombinant proteins was represented as mean (± SD) values.

A specific PCR was performed (data not shown) in which a 428 bp PCR product could represent the presence of the *exoU* gene. To assess the biological activity of the recombinant proteins, inhibition of ExoU + PA strains in RBC lysis was investigated. The deionized water was used as the positive control representing the highest RBC hemolysis rate. RBCs treated with ExoU + PA strain represented 87% hemolysis which was significantly inhibited after treatment with the two expressed recombinant proteins (41 and 43% hemolysis for YFL001 and YFL002, respectively) (Fig. 9).

#### Inhibition of PA induced cellular cytotoxicity

**Figure 10:**
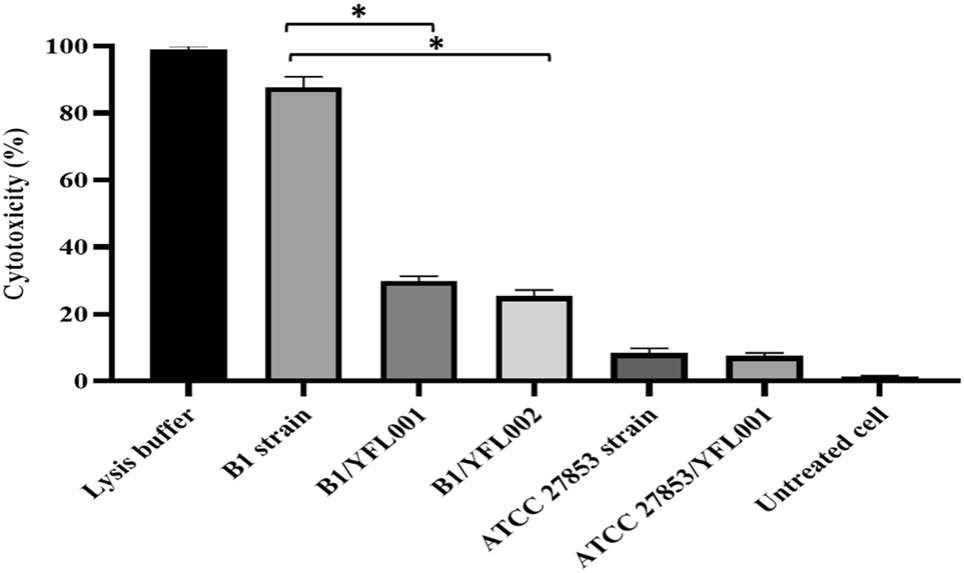
Cellular cytotoxicity of ExoU- and ExoU + PA strains in the presence and absence of recombinant proteins. The observed cellular cytotoxicity was represented as mean (± SD) values. *P* values less than 0.05 were designated with asterisks.

In order to assess the effect of the recombinant proteins in inhibiting cellular cytotoxicity of ExoU + PA strains, the released lactate dehydrogenase was measured following treatment of A549 cells with PA strains alone as the control group and PA strains treated with the recombinant proteins. The results showed the inhibitory effect of the recombinant proteins against ExoU + PA cellular cytotoxicity as the amount of released lactate dehydrogenase was significantly decreased in A549 cells treated with PA strain plus antibodies (YFL001: 31%; YFL002: 27%) in comparison to the control A549 cells treated with lysis buffer (100%) or ExoU + PA strain alone (84%) (Fig. 10).

### Serum stability of the recombinant proteins

**Figure 11:**
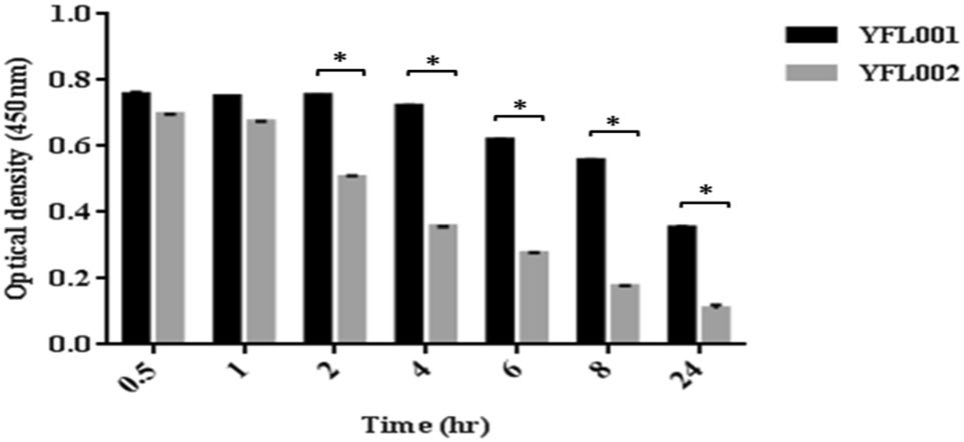
Analysis of serum half-life of recombinant proteins. The observed protein serum half-life was represented as mean (± SD) values. *P* values less than 0.05 were designated with asterisks.

In order to investigate serum stability of the recombinant proteins, a home-made ELISA test was developed to measure this parameter following serum-protein incubation at 37 °C during different times (0.5 h to 24 h). The results showed that YFL001, the scFv molecule attached to the mCH3 fragment, was more stable in serum which was significantly different at longer incubation times (Fig. 11).

## Discussion

Despite development of anti-pseudomonas chemical agents, PA infection is still considered a life-threatening risk factor especially in immunocompromised patients due to the inherent and acquired antibiotic resistance in which low permeability of the bacterial outer membrane, the presence of drug extrusion pumps, and occasionally the production of beta-lactamases^6,7^. During the last decades, alternative anti-PA active or inactive immunotherapy approaches have targeted several bacterial virulence factors including LPS, alginate, extracellular proteins, exotoxin A, and flagellum^22,23^. Previous studies have shown that the Type III secretion system (T3SS) and particularly PcrV protein have major roles in PA pathogenicity through direct transfer of bacterial toxins (ExoY, ExoS, ExoT, ExoU) to the eukaryotic cells^24,25^ and *P. aeruginosa* mutants deficient in PcrV gene are not able to transfer their cytotoxins and consequently their pathogenicity is significantly reduced^6,26^. Thus, anti-PcrV antibodies are assumed to help in reduced PA pathogenicity and clearance of the infection^6,26,27^.

High cost in development of monoclonal antibodies has limited the application of these reagents in microbial treatment approaches. A number of mAbs and antibody fragments are in various stages of development against the PcrV protein^23^. In 2002, the first anti-PcrV mAb, IgG2b type MAb166, could neutralize PA infection in animal models through systemic or intratracheal administration^9,28^. This antibody in its full size or Fab format showed therapeutic as well as prophylactic effects indicating that the Fc fragment is not required in protection against bacterial infection^9,28^. In 2009, Baer et al., designed a humanized anti-PcrV Fab antibody fragment (Fab 1A8) which competes with Mab166 in binding to similar epitopes on the PcrV molecule^11^. This Fab molecule demonstrated T3SS neutralizing activity in in vitro cytotoxic assays as well as in vivo animal studies protecting mice against lethal doses of PA. All these molecules represented a concentration dependent antimicrobial activity yielded in normal body temperature and bacterial clearance^29^. In order to increase serum half-life of this Fab fragment, polyethylene glycol (PEG) was chemically conjugated to the molecule^23^. The PEGylated Fab molecule, KB001-A, originating from murine Mab166, represented T3SS blocking effects in in vitro experiments and required higher concentrations for protection in animal models. Although no serious adverse events were reported for the PEGylated Fab molecule during the first phase of the clinical trials in CF patients suffering from chronic lung infections, the Phase II clinical trial on 182 cystic fibrosis patients failed to prevent the disease progression^29^.

In 2014, Warrener et al., screened a panel of newly developed anti-PcrV antibodies to find more potent antibodies with greater T3SS inhibitory properties^13^. The two Fab antibody fragments, 29D2 and V2L2MD derived from immunized mice, provided protection against PA infection in animal models. T3SS inactivation cellular assays and animal model experiments showed V2L2MD as a more efficient therapeutic agent in comparison to the MAb166 antibody molecule. In the present study, anti-PcrV scFv antibody fragment was originated from V2L2 Fab molecule^13^. Despite easy engineering of scFv molecules due to their low molecular weight (~ 28 kDa) and their wide range of therapeutic and diagnostic applications, these molecules display a short half-life in comparison to the full-size antibodies. CH2 and CH3 domains of immunoglobulins have been well documented in the interaction with FcRn^18,30^. A successful generation of single-engineered CH3 monomeric (mCH3) in soluble format was reported in 2013^18^ which bound to pH-dependent FcRn similar to the whole antibody molecule. In the next step, engineered mCH3 possessing an additional disulfide bond (mCH3cc) yielded in increased thermal stability of the molecule without affecting its FcRn binding. The engineered mCH3cc domain is a good candidate for increasing the half-life along with better tissue penetration, and ultimately increasing therapeutic efficacy for the fusion components.

In order to examine the role of mCH3cc domain in increasing the stability of the anti-PcrV scFv molecule, this domain was genetically fused to the scFv encoding sequence and structural properties and biological activities of the expressed proteins were compared in in vitro experiments.

In the present study, first, we investigated computationally the conformational stability of the recombinant proteins by prediction of the tertiary structures. Structural validation of the predicted models represented the high quality of the final YFL001 and YFL002 models. Furthermore, these proteins showed similar physiochemical properties in terms of pI and hydropathy values.

Next, the interaction pattern of the scFv molecules towards PcrV antigen was evaluated by molecular docking. Ligplot + analysis indicated that residues R159, E227, E231, and K232 of the PcrV and Y63 and H110 of CDRs of the scFv were common in these two complexes and the residual interface of the CDRs in complex with PcrV showed slight differences. Finally, the structural stability and solvent accessibility of the complexes were evaluated during 100 ns MD simulations. The YFL001/PcrV complex displayed not only the minimum structural deviation during the simulation but also fewer changes were shown in the radius of the gyration plot that signified the well folded structure of YFL001 in the presence of mCH3cc sequence. Similar residual fluctuations were also detected in YFL001 and YFL002 anti-PcrV scFv molecules even with slightly higher fluctuations than the mCH3cc sequence. Furthermore, similar free energy of binding and binding affinity of YFL001 and YFL002 in the interaction with the PcrV antigen ruled out the effect of mCH3cc sequence in the scFv-PcrV interaction. The computational findings in regard to the higher stability of the scFv protein fused to the mCH3cc fragment were reconfirmed through the in vitro serum stability test and it was shown that the addition of the mCH3cc domain could enhance serum stability of the scFv molecule.

Comparison of the spectra obtained from CD analysis showed that despite the presence of approximately 260 amino acids in common, these two r-proteins underwent secondary structural rearrangements resulting in a short peak at 197 nm in YFL002 (similar to the β-sheet structures) which was absent in YFL001. The assessment of tertiary structures by CD spectroscopy was quite a qualitative assessment due to the unknown number of chromophores of these proteins.

The expressed scFv molecules exhibited inhibitory effects on the red blood cell lysis induced by cytotoxic (ExoU positive) PA strains in accordance to the previously published observations about V2L2 antibodies. This phenomenon can be explained by the blockage of the PcrV protein by the scFv molecule irrespective to the mCH3 sequence. In another experiment, these two recombinant antibodies could inhibit the release of the LDH by ExoU positive PA strains in a cell-based toxicity assay confirming the suppression of the PcrV protein which was not observed when ExoU negative PA strains were tested. Taken together these in vitro experiments supported the bioinformatics findings and revealed that the addition of the mCH3cc domain did not alter the biological activity of the scFv antibody fragment similar to the findings Ying et al. reported^18,30^.

## Conclusions

Antibody fragments including Fab and scFv molecules with binding activity of whole antibodies may be considered as the next antibody therapeutics. Although their higher penetration into the solid tissues makes them attractive agents, their reduced half-life has been considered as a drawback. Therefore, optimization of the size and serum half-life of these antibody fragments is necessary. In the present study, an anti-PcrV scFv antibody molecule was engineered from a previously reported potential Fab antibody and the obtained results suggested that the addition of the mCH3cc domain can be a suitable and efficient approach for increasing the stability of the scFv molecule without alteration of its biological activity. The importance of the in vivo testing of this recombinant antibody fragment is also considered.

## Materials and methods

### Bacterial strains

*Escherichia coli* TOP10F’ and BL21 (DE3) strains have been used in this study. Eighteen *P. aeruginosa* strains isolated from the wounds of burnt patients were provided by Dr. Abdi-Ali (Alzahra University, Tehran) in order to determine *exoU* positive strains. Two *exoU* negative PA strains (PAO1 and ATCC27853) were also provided by the Microbiology Department of Pasteur Institute of Iran.

### Design of the expression cassettes

The gene fragment encoding scFv molecule (YFL) was extracted from the published patent (US20150023966A1) and subcloned into the pET28a expression vector (Novagen, USA) after *E. coli* codon optimization*.* The YFL001 gene fragment composed of anti-PcrV scFv genetically conjugated to the published mCH3cc sequence^13^ in order to increase serum stability of the protein while the YFL002 fragment contains the scFv sequence alone.

### Bacterial genotyping studies

Bacterial genomic DNA was extracted by a boiling method. In brief, a colony of each PA isolate was inoculated into 5 ml liquid YT medium and incubated at 37 °C overnight in a shaker. The bacterial pellet was resuspended in NaOH solution (50 mM) and incubated in a 100 °C water bath for 20 min. Tris–HCl (1 M, pH7.5) was added to the samples and the suspension was centrifuged at 3000 rpm for 5 min. The supernatant was isolated as semi-purified genomic DNA. ExoU genotyping was performed through specific PCR using primer sequences extracted from the literature (F: 5′-gggaatactttccccgggaagtt-3′; R: 5′-cgatctcgctctctaatgtggtt-3′)^31^.

### Modeling, refinement and quantitative evaluation of the proteins

The amino acid sequence of anti-PcrV heavy and light chains’ variable regions were linked together through a 15 amino acid linker ((Gly_4_Ser)_3_) to construct the YFL002 cassette (VH-(Gly_4_Ser)_3_ linker-VL-Histidine tag). The recombinant YFL001 protein was oriented as YFL002-(Gly_4_Ser)_3_ linker-mCH3cc-Histidine tag through genetic conjugation of the mCH3cc encoding sequence to the C-terminus of the YFL002 molecule. The amino acid sequences of the two scFv molecules were submitted to HHpred (RRID: SCR_010276) to establish the template-based homology modeling^32^. The crystal structure of the heavy and light chains of the anti-PcrV Fab molecule with 2.78 Å resolution was retrieved from the existing crystal structure (6CYF.LM) in the RCSB Protein Data Bank (PDB). This template protein resulted in 95% identity and 1.48% similarity with YFL002. Furthermore, the crystal structure of human IgG1 Fc fragment with 1.52 Å resolution (5HSF.A) as the template of mCH3cc showed 78% identity and 1.12% similarity, respectively. Comparative protein structure modeling was performed by the MODELLER software 9.19 (RRID: SCR_008395) as a computing system^33^. Top predicted models were selected based on the Discrete Optimized Potential Energy (DOPE) Score of the structure evaluations from the ranked 10,000 generated models. The stereochemical quality of the protein structures were analyzed utilizing SAVES web tools^34,35^ and the structural similarity between each model and the template was evaluated using the TM-align web server^36^. Finally, the best model was selected and considered for further analysis.

### In silico evaluation of the recombinant proteins

The physicochemical properties of YFL001 and YFL002 including estimated half-life, instability index as well as grand average hydropathicity (GRAVY) were calculated by the ProtParam tool in the ExPASy webserver^37^. Prediction of the protein half-life in ProtParam relies on the N-end rule (the identity of its N-terminal residue) within human, yeast and *E. coli* organisms^38,39^. The instability index estimates the in vivo stability of a protein from its amino acid sequence, where values lower than 40 indicate stable proteins^40^. The GRAVY value of a protein is calculated by the sum of hydropathicity of all its amino acids divided by the protein length^41^.

### YFL001/YFL002-PcrV in silico interaction analysis

The tertiary structure of PA PcrV protein was obtained from PDB: 6CYF.I. The docking studies were carried out using the HADDOCK (High Ambiguity Driven protein–protein DOCKing) web server to find the interaction pattern of YFL001 and YFL002 molecules towards PcrV protein^42^. The complementarity determining regions (CDRs) of the anti-PcrV molecules were defined as the active sites of YFL001 and YFL002 (S35, Y36, A37, M38, N39, A54, I55, T56, I57, S58, G59, I60, F61, A62, Y63, Y64, T65, D66, S67, V68, K69, G70, E103, E104, F105, L106, P107, G108, T109, H110, Y111, Y112, Y113, G114, M115, D116, V117, R172, A173, S174, Q175, G176, I117, R178, N179, D180, L181, G182, S198, A199, S200, F201, L202, Q203, S204, L237, Q238, D239, Y240, Y242, P243, W244, T245) (US20150023966A1). PcrV amino acid residues S157, R159, A166, P184, D188, G203, E227, E231, K232, D233, G238, and D246 were considered as the active residues in molecular docking^43^. Visualization and analysis of the complexes originated from the top HADDOCK cluster was performed using PyMol (RRID: SCR_000305) and LigPlot + software (RRID: SCR_018249), respectively^44,45^.

### Molecular dynamics simulation

Protein conformational changes and the stability under physiological conditions of the models were evaluated by molecular dynamics (MD) simulation using GROMACS 2018 package (RRID: SCR_014565) with all-atom optimized potentials for liquid simulations (OPLS-AA) force field^46^. The system was generated by solving the proteins in a cubic box with 1 nm distance from the edges and the simple point charge (SPC) as the water model during all simulations^47^. The net charge of the system was neutralized with the proper number of Cl^-^ and Na^+^ ions. The system energy minimization was conducted by taking advantage of steep-descent with a tolerance of 10 kJ/mol/nm in 500,000 steps and the periodic boundary condition in x, y, and z directions. It was equilibrated in the canonical and isothermal-isobaric ensembles using the Verlet algorithm with an integration time step of 0.01 ps. A 1.2 nm cut-off was set for Coulomb interactions and the particle mesh ewald (PME) method was applied for long-range electrostatic calculations^48^. Final equilibrated systems were subjected to MD simulations for 100 ns at 300 K with a 2 fs time step. Comparative analysis of the time-dependent properties was performed for the trajectories according to the root mean square deviation (RMSD), root mean square fluctuation (RMSF), radius of gyration (RoG), number of hydrogen bonds (H-bonds) in the interface of proteins and surface accessible surface area (SASA). The free energy of binding (ΔG) and dissociation constant (K_d_) were computed using the PRODIGY web server^49^. The graphs were presented using GraphPad Prism version 7 (USA).

### Expression of YFL001 and YFL002 proteins

Expression vectors were transformed into *E. coli* BL21 (DE3) strains. Bacterial single colonies were inoculated into 5 ml LB broth medium supplemented with 30 µg/ml kanamycin. Expression of the proteins was induced by the addition of 0.5 mM IPTG when the optical density of the medium at 600 nm reached 0.4 or 0.5. After 4 h incubation at 37 °C, the bacterial pellet was harvested by centrifugation and protein expression was analyzed on 12% SDS-PAGE stained with Coomassie Blue dye.

### Immunoblotting

Bacterial lysates were run on 12% SDS-PAGE under non-reduced condition and proteins were transferred to a nitrocellulose membrane (Amersham, USA). After blocking the membrane with 3% BSA in phosphate buffered saline (PBS) for 1 h, the membrane was washed 3 times with washing buffer (PBS supplemented with 0.05% Tween20) and incubated with 1:1000 dilution of anti-histidine HRP conjugated antibody (Roche) for an additional 2 h at room temperature. After the washing step, 3,3′ diaminobenzidine tetrahydrochloride (DAB) solution was added to visualize the desired bands.

### Protein purification by affinity chromatography

Protein expression was performed in 500 ml LB broth medium and the bacterial pellet was resuspended in lysis buffer (10 mM Tris base, 100 mM NaH_2_PO_4_, 8 M Urea; pH8.0) and lysed through ultrasound sonication (12 times with 20 s pulses and 20 s rest intervals). Following centrifugation at 6000 rpm (2 min), the supernatant was loaded on Ni–NTA agarose column (QIAGEN) equilibrated with the same lysis buffer. After the washing step (10 mM Tris base, 100 mM NaH_2_PO_4_, 8 M Urea; pH6.3), protein was eluted using the elution buffer (10 mM Tris base, 100 mM NaH_2_PO_4_, 8 M Urea, pH4.5) and urea was gradually removed from the protein solutions within 4 days through dialysis at 4 °C.

### Dynamic Light Scattering analysis

To detect the polydispersity (size spread) of the recombinant antibody fragments, dialyzed samples were tested by Dynamic light scattering (DLS). DLS technique determines the size spread of particles as polydispersity (PDI) index^50^. 500 µg/ml of refolded r-proteins were filtered and analyzed by Zetasizer system (ZEN3600). The results were expressed in terms of the Z-average in nanometer.

### Structural analysis of the recombinant proteins

To compare the secondary structure (alpha helix, beta structures, and random screws) of the two recombinant proteins and evaluation of the structural effects of mCH3 sequence, far-UV (190–250 nm) circular dichroism (CD) spectrometry at 25 °C was performed. In brief, lyophilized protein samples were resuspended in injectable deionized water (pH7.4) to a final concentration of 0.2 mg/ml and analyzed in the Jasco J-810 Spectropolarimeter (Japan) and a far-UV CD spectrum was generated. To analyze changes in protein tertiary structures, a near-UV (250–350 nm) CD spectrum was used at 25 °C. The CD-spectra were analyzed using the program Spectra Manager for Windows 95/NT, Spectra Analysis, Version 1.53.02 [Build 1], JASCO Corporation^51,52^. To investigate the structural similarity of the two expressed proteins, hydrogen (proton) nuclear magnetic resonance spectroscopy (H-NMR) was used. Lyophilized 15 mg of each protein sample with a final concentration of 1 mg/ml was resolved in D_2_O (Heavy water) and tested in the INOVA 300 MHz device (Tarbiat Modares University, Tehran).

### Assessment of the biological activity

#### RBC lysis inhibition assay

*ExoU* gene positive and negative PA strains were inoculated in 5 ml YT broth medium. Human RBCs were washed in PBS (pH7.0). Bacteria and the recombinant proteins were diluted in PBS according to the published protocols (5 × 10^8^ cfu/well and 160 µg/ml, respectively)^22,53^. One hundred microliter of 2% RBC solution was added to the 96-well microplates; v-shaped bottom (Citotest, China). A solution of 100 µl of diluted bacteria was added to each well, and distilled water and PBS were used as hemolysis positive and negative controls. The plates were incubated for 4 h at 37 °C and then centrifuged at 4000 rpm for 5 min. The supernatant of each well was transferred to the flat bottom plate and the optical absorption was measured at 405 nm by the ELISA reader (Biohit, Finland). Ethics Committee of Institute Pasteur of Iran approved the study in 2019.

#### Cell lysis inhibition assay

SKBR3 (Human breast cancer cell line) (RRID: CVCL_0033) and A549 (Human non-small cell lung cancer cell line) were used as control and test cell lines, respectively. According to the CytoTox-One kit (Promega) instructions, 2 × 10^4^ cfu/well mammalian cells were needed for this test. In addition, to obtain the multiplicity of the infection (MOI) ratio of 10^21^, 2 × 10^5^ cfu/well bacteria (from B1 and ATCC27853 strains) were prepared in PBS. Bacteria were added to the cell lines in a culture plate and incubated for 2 h at 37 °C under 5% CO_2_. Then, the CytoTox-One reagent was added to each well and incubated for 10 min. The reaction was stopped with stop solution and the plate contents were transferred to a black polystyrene plate and the optical absorption was read at 560 and 590 nm excitation and emission wavelengths by the fluorometer (Biotek). Released lactate dehydrogenase (LDH) from the damaged cells was quantified according to the kit instructions.

### Serum stability determination

In order to assess protein stability, recombinant proteins (YFL001 and YFL002) were diluted to 75 µg/ml with freshly isolated human serum samples and incubated at 37 °C for 30 min, 1 h, 2 h, 4 h, 6 h, 8 h, and 24 h. At the time points mentioned above, 100 µl aliquots were taken and analyzed in a homemade ELISA assay.

In brief, 5 µg/ml bacterial lysate (Originated from the two *exoU* positive and negative PA strains, B1 and ATCC27853, respectively) was coated in ELISA microplates (Overnight at 4 °C). After blocking the wells with 1% BSA in PBS, the serially diluted protein samples mentioned above were added to the corresponding wells and incubated for 2 h at room temperature. Mouse HRP-conjugated anti-Histidine antibody (1:750 dilution) was added to the wells and incubated for 1 h at RT. TMB substrate was added to each well and the reaction was stopped after 10 min with 2 M H_2_SO_4_ and optical absorbance was measured at 450 nm using the ELISA reader (Biohit).

### Ethics approval

Serum stability study was accomplished in accordance with the declaration of Helsinki ethical principles and confirmed by the Ethics Committee of Pasteur Institute of Iran (IR.PII.REC.1399.012). Fresh serum sample was isolated from one of the authors as a volunteer.

## Supplementary Information

Supplementary Information.
