# Supplementary material for: Characterization of a novel mCH3 conjugated anti-PcrV scFv molecule
Source: Sci Rep. 2021 Mar 30;11:7154. doi: 10.1038/s41598-021-86491-w (PMC8010009; doi:10.1038/s41598-021-86491-w)
Supplement: Supplementary file 1 — Supplementary Information. [file 41598_2021_86491_MOESM1_ESM.docx]

**Characterization of a novel mCH3 conjugated anti-PcrV scFv molecule**

Samira Komijani^a, b*^, Elham Bayat^b, c*^, Elham Rismani^d^, Soma Hosseini^b^, Reza Moazzami^b^, Leila Nematollahi^b^, Soroush Sardari^b^, Yeganeh Talebkhan^b#^, Fatemeh Davami^b#^, Farzaneh Barkhordari^b^, Fakhrisadat Hosseini^a^, Hoda Jahandar^e, f^

^a^ Department of Biotechnology School of Biology, Alzahra University, Tehran

^b^ Biotechnology Research Center, Pasteur Institute of Iran

^c^ Department of Molecular and Cellular Sciences, Faculty of Advanced Sciences & Technology, Tehran Medical Branch, Islamic Azad University, Tehran

^d^ Molecular Medicine Department, Biotechnology Research Center, Pasteur Institute of Iran, Tehran, Iran

^e^ Pharmaceutical Sciences Research Center, Tehran Medical Sciences, Islamic Azad University, Tehran, Iran

^f^ Department of Basic Sciences, Faculty of Pharmacy and Pharmaceutical Sciences, Tehran Medical Sciences, Islamic Azad University, Tehran, Iran

**Supplementary Figure 1.** **Protein expression analysis**. The full size original image of Figure 5A (YFL001): #1, 2: Lysate of *E. coli* BL21 (DE3) harboring pET28a vector before and after induction (BI, AI); #3, 4, 7: Recombinant *E. coli* lysate before induction (BI); M: Protein Mw marker (Fermentas); #5, 6, 8: Recombinant BL21 *E. coli* lysate after induction (AI).


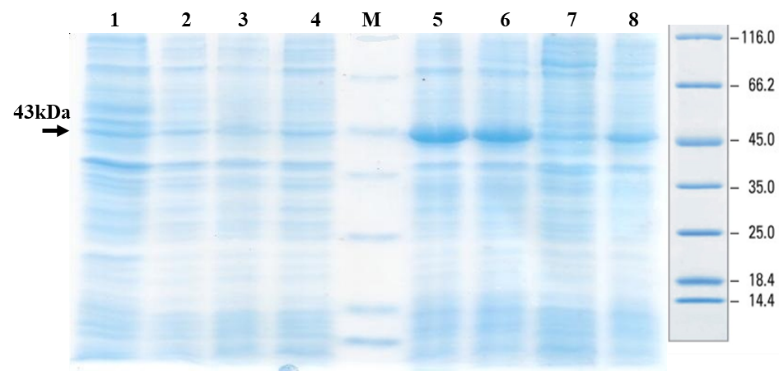


**Supplementary Figure 2.** **Protein expression analysis**. The full size original image of Figure 5B: #1, 2: Recombinant YFL001 *E. coli* lysate after induction (AI); #3, 4: Recombinant YFL002 *E. coli* lysate after induction (AI); #5: Recombinant YFL002 *E. coli* lysate (BI).

^
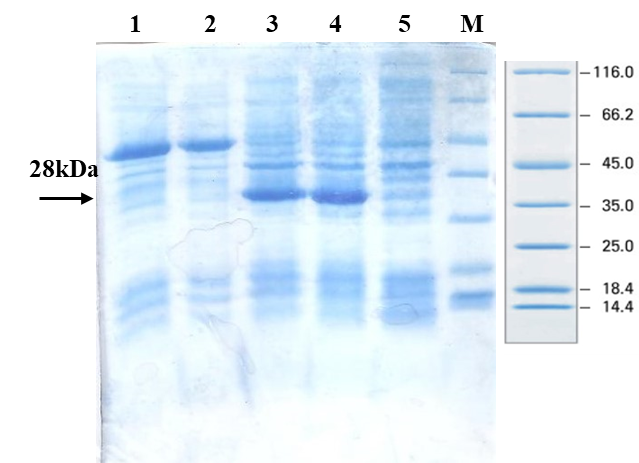
^

**Supplementary Figure 3.** **Protein expression analysis**. The full size original image of Figure 5C (Non-reduced Western blotting analysis): M: Protein Mw marker (Thermo Fisher Scientific); #1, 2: YFL001 and YFL002 expressing recombinant *E. coli* lysates (AI); #3: His-tagged 12 kDa recombinant protein (positive control).


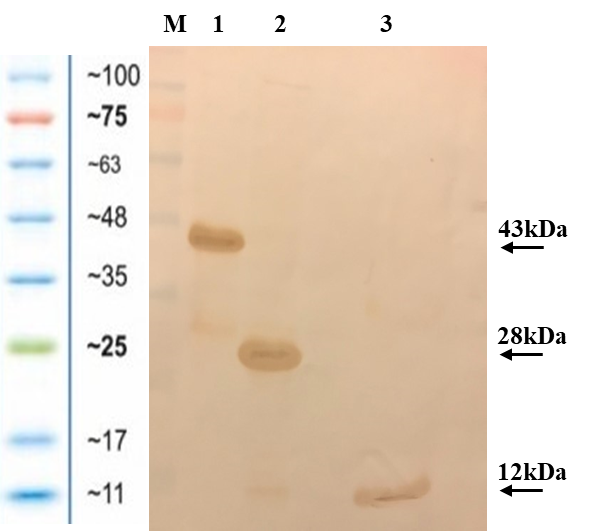


**Supplementary Figure 4.** **Protein purification**. The full size original image of Figure 6A (YFL001): #1, 2: Recombinant *E. coli* lysate (BI and AI); M: Protein Mw marker (Fermentas); #3: Initial sample; #4-8: Eluted protein samples.


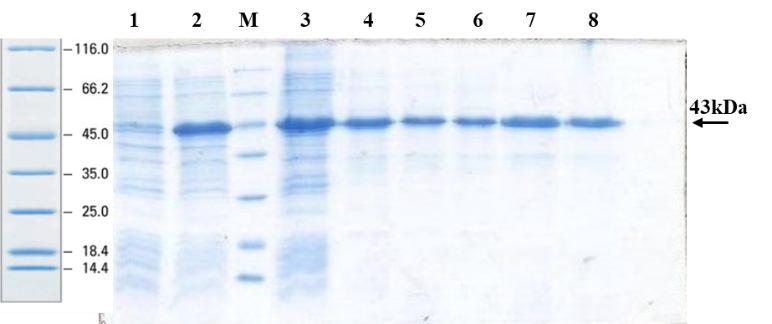


**Supplementary Figure 5. Protein purification.** The full size original image of Figure 6B (YFL002): #1, 2: Recombinant *E. coli* lysate (BI and AI); M: Protein Mw marker (Fermentas); #3: Flow through sample; #4-7: Eluted protein samples.

*
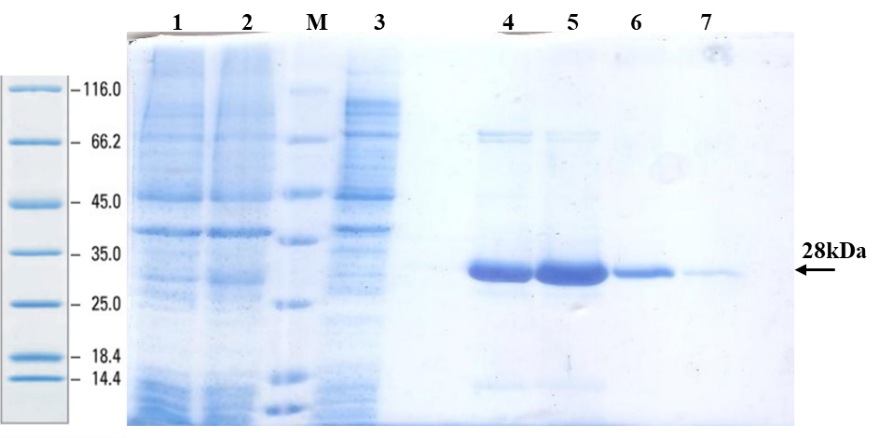
*
